# Association between Allogeneic or Autologous Blood Transfusion and Survival in Patients after Radical Prostatectomy: A Systematic Review and Meta-Analysis

**DOI:** 10.1371/journal.pone.0171081

**Published:** 2017-01-30

**Authors:** Su-Liang Li, Yun Ye, Xiao-Hua Yuan

**Affiliations:** 1 Department of Laboratory Medicine, The First Affiliated Hospital of Xi’an Medical University, Xi’an, Shaanxi, China; 2 Department of Blood Transfusion, The First Affiliated Hospital of Xi’an Medical University, Xi’an, Shaanxi, China; University of Kentucky, UNITED STATES

## Abstract

**Background:**

A number of studies have investigated the effect of perioperative blood transfusion (PBT) for patients after radical prostatectomy (RP), with some reporting conflicting results. A systematic review of the literature and a meta-analysis were conducted to explore the association between PBT (autologous or allogeneic) and biochemical recurrence-free survival (BRFS), overall survival (OS) and cancer-specific survival (CSS) in patients undergoing RP.

**Methods:**

The PubMed, Medline, Cochrane Library, and Embase databases were searched for published controlled clinical studies on perioperative allogeneic or autologous blood transfusion (BT) and patient survival after RP. STATA software version 12.0 was used for data analysis. We used hazard ratios (HRs) and 95% confidence intervals (CIs) to test the correlation between BT and patient survival after RP.

**Results:**

Data from a total of 26,698 patients in ten published studies were included in the meta-analysis. The meta-analysis results showed that autologous BT was not associated with BRFS (HR: 1.06; 95% CI: 0.96–1.18; Z = 1.17; P = 0.24), OS (HR: 0.86; 95% CI: 0.71–1.04; Z = 1.58; P = 0.11), or CSS (HR: 0.98; 95% CI: 0.49–1.96; Z = 0.05; P = 0.96). Allogeneic BT exhibited a significant association with worse BRFS (HR: 1.09; 95% CI: 1.01–1.16; Z = 2.37; P = 0.02), OS (HR: 1.43; 95% CI: 1.24–1.64; Z = 4.95; P<0.01) and CSS (HR: 1.74; 95% CI: 1.18–2.56; Z = 2.81; P = 0.005).

**Conclusion:**

Our data showed an association between allogeneic BT and reduced BRFS, OS and CSS in patients after RP. These findings indicate that perioperative blood conservation strategies are important for decreasing the allogeneic BT rate.

## Introduction

Prostate cancer (PCa) is a common malignant tumor of the male urogenital system, the second leading cause of cancer mortality in men worldwide and a significant cause of death in elderly men [1,2]. Radical prostatectomy (RP) is a standard treatment for clinically localized PCa [3,4]. Although many patients are disease free after surgery, many patients still continue to experience PCa recurrence. RP is associated with increased blood loss, which may lead to a need for either autologous or allogeneic transfusion [5]. Blood transfusion (BT) can be lifesaving in the perioperative period, but there are potential risks that can be attributed to transfusion-transmitted infection and transfusion-related immunomodulation (TRIM) [6].

Previous studies have shown that BT has an adverse effect on patient survival in different cancers [7,8]. For example, a relationship between autologous or allogeneic transfusion and the recurrence of PCa has been reported [9,10]. Nonetheless, other studies have reported inconsistent conclusions [11,12]. To obtain most conclusive results, we conducted a systematic review of the literature to explore the association between perioperative BT (PBT; autologous or allogeneic) and biochemical recurrence-free survival (BRFS), overall survival (OS) and cancer-specific survival (CSS) in patients undergoing RP.

## Materials and Methods

### Identification and eligibility of relevant studies

This meta-analysis was carried out in accordance with the Meta-Analysis of Observational Studies in Epidemiology guidelines [13]. A comprehensive search of the literature in the PubMed, Medline, Cochrane Library and Embase databases up to August 30, 2016, was performed using the following keywords: (“transfusion”, “blood transfusion” OR “metachysis”) and (“prostate cancer”, “PCa”, “prostatic neoplasm”, “prostate carcinoma”, “cancer of the prostate” OR “prostatic cancer”) and (“radical prostatectomy”). Only publications written in English with available full text were included in this meta-analysis. The identified literature was reviewed to ensure that the content included the required information. Survival outcomes sought by the search strategy included BRFS, OS and CSS. The following study inclusion criteria were used: (1) the study had to report on the correlation between PBT and survival in patients undergoing RP, (2) data on survival outcomes had to be available, and (3) the full text of the article had to be available and in English. Meanwhile, the exclusion criteria were as follows: (1) studies not focused on transfusion; (2) studies not reporting relevant survival data; (3) studies not published in English; and (4) animal studies, reviews, comments, letters without original data, duplicated studies and irrelevant articles.

### Data extraction

Two investigators (Yun Ye and Su-liang Li) independently extracted the data from all eligible publications. Any controversy was resolved by discussion with the third investigator (Xiao-Hua Yuan) to adjudicate the disagreement. The following items were extracted from the included studies: the first author’s name; the publication year and country; the recruitment period; the number of allogeneic or autologous BTs; quality scores; and the hazard ratios (HRs) for BRFS, OS and CSS and their 95% confidence intervals (CIs). The HRs were extracted from survival curves when the data could not be obtained directly. All authors agreed on the aspects of the literature to be ultimately considered.

### Quality assessment

According to the Newcastle-Ottawa Scale (NOS) guidelines [14], two independent authors (Yun Ye and Su-Liang Li) evaluated the quality of the included retrospective studies. The scale focuses on three factors: (1) subject selection: 0–4, (2) inter-subject comparability: 0–2, and (3) exposure: 0–3. The total score ranges from 0 (lowest) to 9 (highest). We identified “high-quality” choices as those with scores of 6–9, whereas scores of 0–5 are considered to indicate poor quality [15]. A third reviewer was consulted when there were disagreements on the NOS scores of the studies between the two authors, and our meta-analysis only enrolled high-quality studies.

### Statistical analysis

A forest plot was used to aggregate HRs and 95% CIs from individual studies to obtain a summary HR of the effect of BT. Heterogeneity was analyzed using the Cochran’s *Q*-statistic test, and P≤ 0.05 was considered as statistically significant [16]. If the statistical power of Cochran’s *Q*-statistic was low, the I^2^ test was also used to evaluate heterogeneity among studies (values of 25%, 50% and 75% were considered to represent low, medium, and high heterogeneity, respectively) [17]. We pooled the results using random-effects models because the CI for the average intervention effect would be wider and corresponding claims of statistical significance would be more conservative [18]. Sensitivity analysis was conducted to validate the credibility of outcomes through the omission of individual studies from the meta-analysis. Funnel plots were used to explore whether any obvious publication bias existed. Egger’s linear regression test was used to evaluate the symmetry of the funnel plots [19]. A P-value of 0.05 was regarded as significant, and all tests were two sided. STATA version 12.0 (Stata Corp LP, College Station, TX, USA) was used to perform statistical analyses.

## Results

### Study selection and characteristics

**Fig 1:**
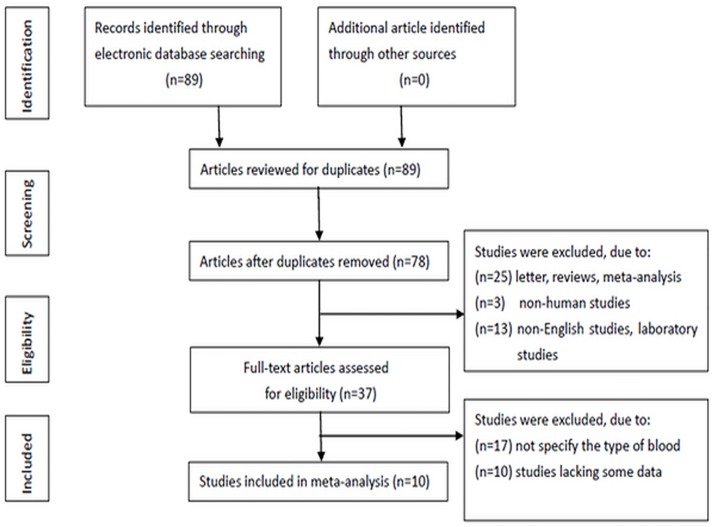
Flow chart showing the study selection procedure.

The details of the study search are presented in a flow chart (Fig 1). In total, 89 relevant studies were identified. After careful reading of each article, 52 studies were excluded because they were duplicates, letters, reviews, meta-analyses, non-human studies, non-English-language studies, or laboratory studies. After the remaining studies (n = 37) were reviewed, additional studies were excluded because blood types were not specified or because relevant data were lacking. Ten retrospective studies were ultimately included in the meta-analysis.

The ten studies were published between 1990 and 2016: five European studies [9,11,20–22], three American studies [12,23–24], and two Asian studies [25,26]. The median follow-up period in all studies ranged from 25.2–122.4 months. A median NOS score of 7 was identified as indicating reliable quality (Table 1).

**Table 1:** Characteristics of the eligible studies in the meta-analysis.

First author	Year	Country	Recruitment Period	Autologous BT (n)	Allogeneic BT (n)	Survival analysis	Quality score
Ford BS	2008	USA	1987–2005	252	117	BRFS	7
Boehm K	2015	Germany	1992–2011	548	445	BRFS, OS	7
Yeoh TY	2014	Singapore	1991–2005	-	379	OS, CSS	8
Gallina A	2007	Germany	1992–2005	205	-	BRFS	6
Chalfin HJ	2014	USA	1994–2012	5,124	258	BRFS, OS, CSS	9
McClinton S	1990	UK	1977–1982	-	71	OS	6
Oefelein MG	1995	USA	1980–1990	62	153	BRFS, CSS	7
Eickhoff JH	1991	Denmark	1978–1986	-	60	OS, CSS	6
Paul R	2006	Germany	1984–2003	45	756	BRFS	6
Kim JK	2016	Korea	1993–2014	90	350	BRFS, OS, CSS	8

### Primary outcomes

#### Autologous BT: Meta-analysis of BRFS, OS, and CSS

**Fig 2:**
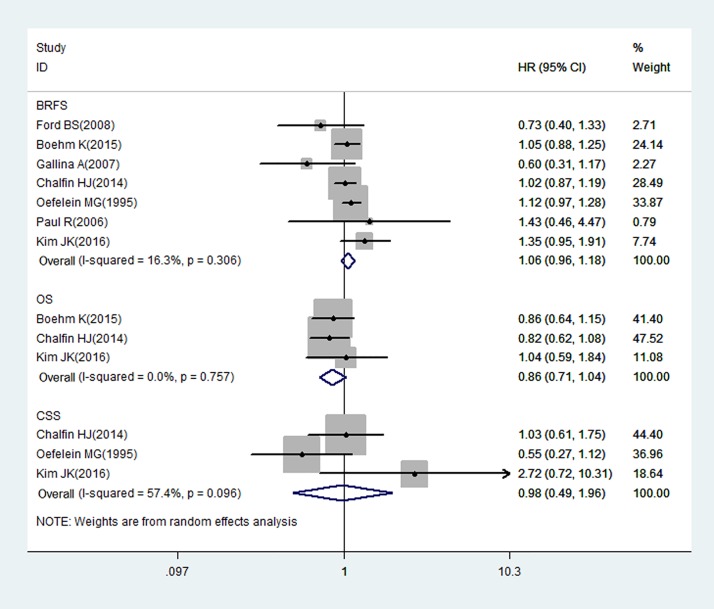
Random-effects model forest plots showing the impact of autologous BT on PCa survival after RP.

Seven studies on autologous BT and survival in patients after RP, which included a total of 15,651 patients, reported BRFS, OS, or CSS as an outcome. Among the patients, 40.4% (n = 6,326) received autologous BTs during the perioperative period. The relationship between autologous BT and the BRFS of patients following RP is illustrated by forest plots in Fig 2. Our results suggest that autologous BT was not associated with BRFS (HR: 1.06; 95% CI: 0.96–1.18; Z = 1.17; P = 0.24) and that heterogeneity among the studies was low (I^2^ = 16.3%, P = 0.31). Three studies provided data on the association between autologous BT and OS. The results of these studies were pooled, and no significant association was found between autologous BT and OS (HR: 0.86; 95% CI: 0.71–1.04; Z = 1.58; P = 0.11), and low heterogeneity was observed among the studies (I^2^ = 0%, P = 0.76). Three studies that evaluated the association between autologous BT and CSS also showed no significant association (HR: 0.98; 95% CI: 0.49–1.96; Z = 0.05; P = 0.96), with moderate heterogeneity among the studies (I^2^ = 57.4%, P = 0.10).

#### Allogeneic BT: Meta-analysis of BRFS, OS and CSS

**Fig 3:**
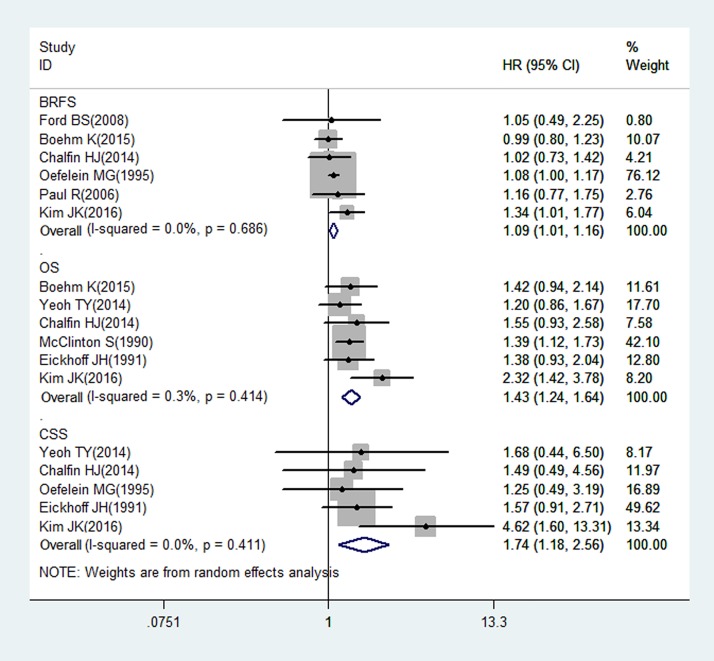
Random-effects model forest plots showing the impact of allogeneic BT on the survival of PCa patients after RP.

Nine studies (n = 15,899) on allogeneic BT and survival in patients after RP reported BRFS, OS, and CSS as outcomes. Among the patients, 16.3% (n = 2,589) received allogeneic BTs. The relationship between allogeneic BT and the BRFS of patients after RP is shown in forest plots in Fig 3. Six studies provided data on the association between allogeneic BT and BRFS. The results of the analysis suggested that allogeneic BT was associated with BRFS (HR: 1.09; 95% CI: 1.01–1.16; Z = 2.37; P = 0.02) and that heterogeneity among the studies was low (I^2^ = 0%, P = 0.69). Six studies provided data on allogeneic BT and OS, and we found a significant association between allogeneic BT and OS (HR: 1.43; 95% CI: 1.24–1.64; Z = 4.95; P<0.01), with low heterogeneity among the studies (I^2^ = 0.3%, P = 0.41). Five studies evaluated the association of allogeneic BT and CSS, and we also found a significant association (HR: 1.74; 95% CI: 1.18–2.56; Z = 2.81; P = 0.005), with low heterogeneity among the studies (I^2^ = 0%, P = 0.41).

#### Sensitivity and publication bias analyses

**Fig 4:**
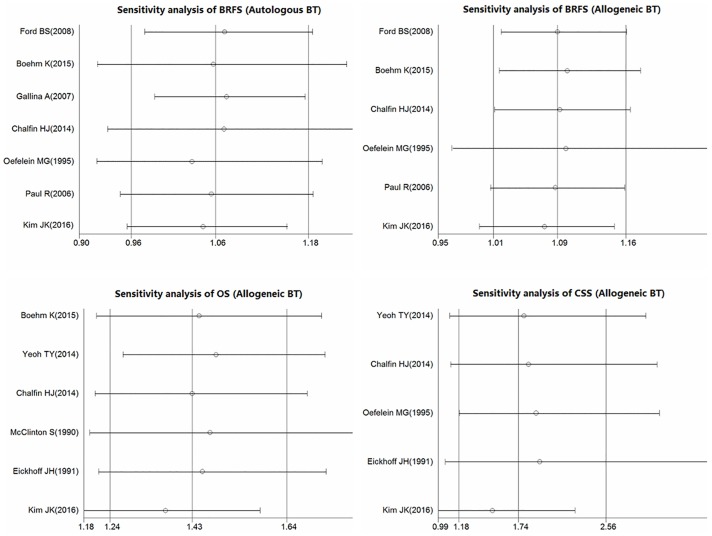
Results of the sensitivity analysis.

**Fig 5:**
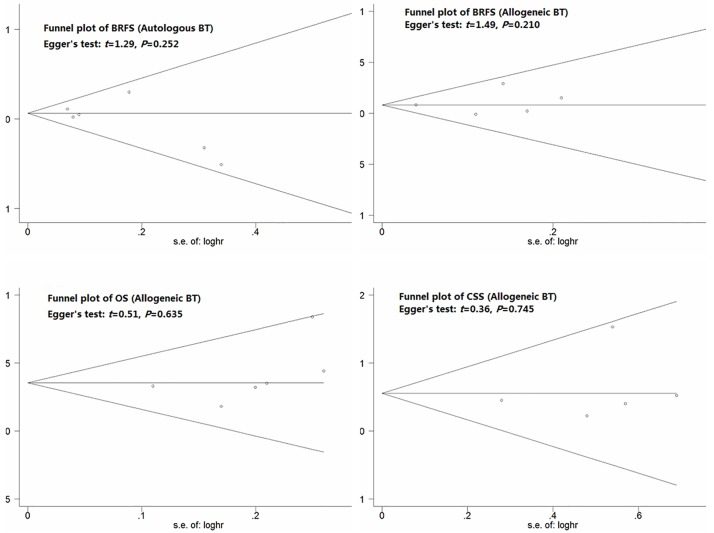
Funnel plot analysis of publication bias.

The overall significance did not change when any single study was omitted. Sensitivity analysis showed that the data were relatively stable and reproducible (Fig 4). The funnel plots of the studies were symmetrical, and Egger’s test showed no publication bias (Fig 5). As only 3 included studies investigated the relationship between autologous BT and OS and CSS, we did not perform publication bias and sensitivity analyses.

## Discussion

By the end of 2016, a projected 180,890 new cases of PCa will have been diagnosed, and 26,120 men will have died of the disease in the United States alone. Mortality from PCa accounts for 8% of all cancer deaths worldwide [27]. RP is the main treatment for clinically localized PCa, which may lead to PBT, and the transfusion rate varies from 1.4–67.0%, depending on the surgical approach used [5]. Although BT is lifesaving and is safer than before, it still poses many significant risks, and the association between PBT and tumors’ clinical outcomes has been under debate over the past few years. Certain studies have demonstrated that BT is an independent risk factor for cancer progress and is associated with decreased survival and increased recurrence of cancer, including lung cancer [28], gastrointestinal cancer [7,29], bladder cancer [8,30], and breast cancer [31].

In fact, several published reports have suggested that PBT has a role in the recurrence of PCa after RP [9,32–33]. Nonetheless, certain studies have reported a contradictory role for PBT in PCa [11,12]. Furthermore, these studies have failed to discuss both autologous and allogeneic BT. Until now, there have been no systematic literature reviews or meta-analyses of the relationship between PBT and patient survival after RP. Therefore, the current meta-analysis was conducted to explore the association between PBT (autologous or allogeneic) and the survival of patients after RP.

The present meta-analysis combined the outcomes of 10 studies published worldwide over the past two decades. Our meta-analysis showed that there was no significant association between autologous BT and BRFS (HR: 1.06; 95% CI: 0.96–1.18; Z = 1.17; P = 0.24), OS (HR: 0.86; 95% CI: 0.71–1.04; Z = 1.58; P = 0.11), or CSS (HR: 0.98; 95% CI: 0.49–1.96; Z = 0.05; P = 0.96). The results demonstrate that autologous transfusion is not a negative predictor of PCa recurrence after RP.

Allogeneic blood is the major cause of TRIM due to constituents that mediate immunosuppression, which is related to allogeneic mononuclear cells, white-blood-cell-derived soluble mediators and soluble HLA molecules circulating in allogeneic plasma [34]. Kim et al. reported that allogeneic PBT during RP was significantly associated with decreased BRFS, OS and CSS in both univariate and multivariate analyses [26]. However, a previous study showed equivalent survival for autologous and allogeneic BT [20]. In our study, allogeneic BT exhibited a significant association with worse BRFS (HR: 1.09; 95% CI: 1.01–1.16; Z = 2.37; P = 0.02), OS (HR: 1.43; 95% CI: 1.24–1.64; Z = 4.95; P<0.01), and CSS (HR: 1.74; 95% CI: 1.18–2.56; Z = 2.81; P = 0.005). Our results support the hypothesis that TRIM occurs in response to allogeneic PBT and suggest that allogeneic BT increases the risk of cancer recurrence and mortality in PCa patients who undergo RP. Therefore, it is important to develop patient-specific blood conservation strategies for the perioperative period to decrease the allogeneic BT rate and improve the clinical outcomes of patients. These strategies should comprise three main components: (i) evaluation of high-risk patients and optimization of erythrocyte mass and function, (ii) minimization of perioperative erythrocyte loss, and (iii) use of patient-specific transfusion triggers to decide when administration of blood products is warranted [35].

Based on the results of the current meta-analysis, we think that allogeneic BT is associated with the prognosis of RP patients. Nevertheless, there are several limitations to the present study. First, there may have been language bias because our study was limited to those studies written in English. Second, the included studies were retrospective. Third, HRs were extracted from survival curves when directly reported HR values were lacking, which may have introduced an element of decreased reliability. Fourth, the sample sizes of the enrolled research studies (from 45–5,124) varied widely, inevitably causing bias to varying degrees. Furthermore, many factors, including preoperative anemia and the blood storage time, might have influenced the oncological outcomes. Further studies are needed to provide the high level of evidence required to address these limitations.

In conclusion, a systematic review of the literature and a meta-analysis showed that allogeneic BT may be associated with reduced BRFS, OS and CSS in patients after RP. This finding provides support for efforts to develop blood conservation strategies and reduce the use of allogeneic PBT in these patients. In addition, the result suggests that to achieve a better outcome for patients after RP, autologous BT represents a safer method that should be recommended. However, a well-designed prospective randomized controlled trial will be required to confirm the safety of autologous BT in patients after RP.

## Supporting Information

S1 PRISMA Checklist PRISMA 2009 Checklist. (DOC)

S1 Appendix Medline (PubMed) search strategy. (DOCX)

S2 Appendix Characteristics of the included studies. (XLS)

S3 Appendix Quality assessment and scoring of nonrandomized studies. (DOCX)

S4 Appendix Quality assessment of studies included in the meta-analysis using a Newcastle-Ottawa Scale. (DOCX)

S1 Data Raw data. (DOCX)
