# Supplementary material for: Association between Allogeneic or Autologous Blood Transfusion and Survival in Patients after Radical Prostatectomy: A Systematic Review and Meta-Analysis
Source: PLoS One. 2017 Jan 30;12(1):e0171081. doi: 10.1371/journal.pone.0171081 (PMC5279775; doi:10.1371/journal.pone.0171081)
Supplement: S1 Appendix — (DOCX) [file pone.0171081.s002.docx]

S2 Appendix. Medline (PubMed) search strategy

#1 transfusion [Mesh]

#2 blood transfusion [tiab]

#3 metachysis [tiab]

#4 #1 OR #2 OR #3

#5 prostate cancer [Mesh]

#6 PCa [tiab]

#7 prostatic neoplasm [tiab]

#8 prostate carcinoma [tiab]

#9 cancer of the prostate [tiab]

#10 prostatic cancer [tiab]

#11 #5 OR #6 OR #7 OR #8 OR #9 OR #10

#12 radical prostatectomy [tiab]

#13RP[tiab]

#14#12 OR #13

#15 #4 AND #11 AND #14
