# Supplementary material for: Association between Allogeneic or Autologous Blood Transfusion and Survival in Patients after Radical Prostatectomy: A Systematic Review and Meta-Analysis
Source: PLoS One. 2017 Jan 30;12(1):e0171081. doi: 10.1371/journal.pone.0171081 (PMC5279775; doi:10.1371/journal.pone.0171081)
Supplement: S3 Appendix — (DOCX) [file pone.0171081.s004.docx]

S4 Quality assessment and scoring of nonrandomized studies.

Note: A study can be awarded a maximum of one star for each numbered item within the Selection and Exposure categories. A maximum of two stars can be given for Comparability.

**Selection**

1) Is the case definition adequate?

a) yes, with independent validation **🟑**

b) yes, eg record linkage or based on self reports

c) no description

2) Representativeness of the cases

a) consecutive or obviously representative series of cases **🟑**

b) potential for selection biases or not stated

3) Selection of Controls

a) community controls **🟑**

b) hospital controls

c) no description

4) Definition of Controls

a) no history of disease (endpoint) **🟑**

b) no description of source

**Comparability**

1) Comparability of cases and controls on the basis of the design or analysis

a) study controls for _______________ (Select the most important factor.) **🟑**

b) study controls for any additional factor **🟑** (This criteria could be modified to indicate specific control for a second important factor.)

**Exposure**

1) Ascertainment of exposure

a) secure record (eg surgical records) **🟑**

b) structured interview where blind to case/control status **🟑**

c) interview not blinded to case/control status

d) written self report or medical record only

e) no description

2) Same method of ascertainment for cases and controls

a) yes **🟑**

b) no

3) Non-Response rate

a) same rate for both groups **🟑**

b) non respondents described

c) rate different and no designation

Available from: URL: http://www.ohri.ca/programs/clinical_epidemiology/ oxford. htm
