# Supplementary material for: Association between Allogeneic or Autologous Blood Transfusion and Survival in Patients after Radical Prostatectomy: A Systematic Review and Meta-Analysis
Source: PLoS One. 2017 Jan 30;12(1):e0171081. doi: 10.1371/journal.pone.0171081 (PMC5279775; doi:10.1371/journal.pone.0171081)
Supplement: S4 Appendix — (DOCX) [file pone.0171081.s005.docx]

**S5. Quality assessment of studies included in the meta-analysis using a Newcastle-Ottawa Scale**

| Study | Selection | Comparability | Exposure | Total score |
| --- | --- | --- | --- | --- |
| Ford BS,2008^[12]^ | **** | * | ** | 7 |
| Boehm K,2015^[20]^ | **** | * | ** | 7 |
| Yeoh TY,2014^[25]^ | **** | ** | ** | 8 |
| Gallina A,2007^[21]^ | *** | * | ** | 6 |
| Chalfin HJ,2014^[23]^ | **** | ** | *** | 9 |
| McClinton S,1990^[9]^ | *** | * | ** | 6 |
| Oefelein MG,1995^[24]^ | **** | * | ** | 7 |
| Eickhoff JH,1991^[22]^ | *** | ** | * | 6 |
| Paul R,2006^[11]^ | **** | * | * | 6 |
| [Kim JK,2016](https://www.ncbi.nlm.nih.gov/pubmed/?term=Kim%20JK%5BAuthor%5D&cauthor=true&cauthor_uid=27159369)^[26]^ | **** | ** | ** | 8 |
