# Supplementary material for: Association between Allogeneic or Autologous Blood Transfusion and Survival in Patients after Radical Prostatectomy: A Systematic Review and Meta-Analysis
Source: PLoS One. 2017 Jan 30;12(1):e0171081. doi: 10.1371/journal.pone.0171081 (PMC5279775; doi:10.1371/journal.pone.0171081)
Supplement: S1 Data — (DOCX) [file pone.0171081.s006.docx]

**S6 Raw data**

**Allogeneic blood：**Test(s) of heterogeneity:

Heterogeneity degrees of

statistic freedom P I-squared** Tau-squared

BRFS 3.09 5 0.686 0.0% 0.0000

OS 5.01 5 0.414 0.3% 0.0001

CSS 3.96 4 0.411 0.0% 0.0000

** I-squared: the variation in ES attributable to heterogeneity)

Significance test(s) of ES=1

BRFS z= 2.37 p = 0.018

OS z= 4.95 p = 0.000

CSS z= 2.81 p = 0.005

**Autologous blood：** Test(s) of heterogeneity:

Heterogeneity degrees of

statistic freedom P I-squared** Tau-squared

BRFS 7.16 6 0.306 16.3% 0.0030

OS 0.56 2 0.757 0.0% 0.0000

CSS 4.70 2 0.096 57.4% 0.2089

** I-squared: the variation in ES attributable to heterogeneity)

Significance test(s) of ES=1

BRFS z= 1.17 p = 0.242

OS z= 1.58 p = 0.114

CSS z= 0.05 p = 0.959

**Autologous blood：**publication bias

BRFS

Egger's test

------------------------------------------------------------------------------

Std_Eff | Coef. Std. Err. t P>|t| [95% Conf. Interval]

-------------+----------------------------------------------------------------

slope | .1170149 .0903601 1.29 0.252 -.1152631 .3492929

bias | -.5536516 .7917135 -0.70 0.516 -2.588816 1.481513

------------------------------------------------------------------------------

**Allogeneic blood：**publication bias

BRFS

Egger's test

------------------------------------------------------------------------------

Std_Eff | Coef. Std. Err. t P>|t| [95% Conf. Interval]

-------------+----------------------------------------------------------------

slope | .071632 .0479544 1.49 0.210 -.0615107 .2047746

bias | .1681059 .5609809 0.30 0.779 -1.389427 1.725639

OS

Egger's test

------------------------------------------------------------------------------

Std_Eff | Coef. Std. Err. t P>|t| [95% Conf. Interval]

-------------+----------------------------------------------------------------

slope | .1156005 .2256197 0.51 0.635 -.5108202 .7420213

bias | 1.43523 1.288587 1.11 0.328 -2.142462 5.012922

CSS

Egger's test

------------------------------------------------------------------------------

Std_Eff | Coef. Std. Err. t P>|t| [95% Conf. Interval]

-------------+----------------------------------------------------------------

slope | .2368367 .6639277 0.36 0.745 -1.876078 2.349751

bias | .7635201 1.505316 0.51 0.647 -4.027068 5.554108

------------------------------------------------------------------------------
